# Differential Expression of PGC-1α and Metabolic Sensors Suggest Age-Dependent Induction of Mitochondrial Biogenesis in Friedreich Ataxia Fibroblasts

**DOI:** 10.1371/journal.pone.0020666

**Published:** 2011-06-07

**Authors:** José Luis García-Giménez, Amparo Gimeno, Pilar Gonzalez-Cabo, Francisco Dasí, Arantxa Bolinches-Amorós, Belén Mollá, Francesc Palau, Federico V. Pallardó

**Affiliations:** 1 Centro de Investigación Biomédica en Red de Enfermedades Raras (CIBERER), Valencia, Spain; 2 Fundación del Hospital Clínico Universitat de Valencia, FIHCUV-Incliva, Valencia, Spain; 3 Instituto de Biomedicina Valencia, Valencia, Spain; 4 Department of Physiology, Medical School, Universitat de València, Valencia, Spain; Brigham and Women's Hospital, Harvard Medical School, United States of America

## Abstract

**Background:**

Friedreich's ataxia (FRDA) is a mitochondrial rare disease, which molecular origin is associated with defect in the expression of frataxin. The pathological consequences are degeneration of nervous system structures and cardiomyopathy with necrosis and fibrosis, among others.

**Principal Findings:**

Using FRDA fibroblasts we have characterized the oxidative stress status and mitochondrial biogenesis. We observed deficiency of MnSOD, increased ROS levels and low levels of ATP. Expression of PGC-1α and mtTFA was increased and the active form of the upstream signals p38 MAPK and AMPK in fibroblasts from two patients. Interestingly, the expression of energetic factors correlated with the natural history of disease of the patients, the age when skin biopsy was performed and the size of the GAA expanded alleles. Furthermore, idebenone inhibit mitochondriogenic responses in FRDA cells.

**Conclusions:**

The induction of mitochondrial biogenesis in FRDA may be a consequence of the mitochondrial impairment associated with disease evolution. The increase of ROS and the involvement of the oxidative phosphorylation may be an early event in the cell pathophysiology of frataxin deficiency, whereas increase of mitochondriogenic response might be a later phenomenon associated to the individual age and natural history of the disease, being more evident as the patient age increases and disease evolves. This is a possible explanation of heart disease in FRDA.

## Introduction

Friedreich Ataxia (FRDA) is an inherited autosomal recessive neurodegenerative disease in which over 96% of patients have a homozygous expansion of a GAA triplet repeat in the first intron of the frataxin (*FXN*) gene on chromosome 9 [1], [2]. The consequence of deficiency on frataxin is that patients have progressive gait and limb ataxia, slurred speech, and peripheral axonal neuropathy associated with hypertrophic cardiomyopathy, diabetes mellitus or glucose intolerance and skeletal deformities. The evolution of the disease leads to severe disability in early adulthood [3], [4].

Pathogenic consequences observed in cells deficient for frataxin include abnormal function of Fe-S clusters (ISC) biogenesis and decreased activities of Fe-S proteins, iron accumulation in mitochondrial matrix, increased reactive oxygen species (ROS) and impairment of the electron transport chain [5], which in turn, leads to decreased ATP production [6]. More recently, a role as an iron sensor involved in the ISC biosynthesis has been proposed for the bacterial frataxin [7]. The hypothesis involving frataxin in the ISC metabolism in mitochondria is the most generally accepted, but the precise sequence of events in FRDA cell pathophysiology still remains elusive.

The relationship between FRDA and oxidative stress seems increasingly clear. This is directly related to disturbances of both iron metabolism and respiratory chain. Iron increases in mitochondrial matrix because of inefficient ISC synthesis and it induces ROS via Fenton chemistry. Moreover, impairment of respiratory chain complexes I, II, and III also induces accumulation of ROS in frataxin defective cells. Previous studies suggest that frataxin might detoxify ROS via activation of glutathione peroxidase and elevation of thiols [8]. FRDA is characterized by a reduction of free glutathione levels in the blood of patients, although total blood glutathione levels were not affected, suggesting extensive glutathionylation of proteins [9]. Additionally, antioxidant cell response is reduced in frataxin-deficient cells being especially relevant the failure to induce the mitochondrial superoxide dismutase, MnSOD. In patients decreased plasma free glutathione, increased plasma levels of the lipid peroxidation product malondialdehyde, and increase urinary 8-hydroxy-2′-deoxyguanosine (a marker of oxidative DNA damage) support evidence of oxidative stress associated with frataxin deficiency [10].

ROS induce damage to mitochondrial DNA (mtDNA) and proteins, and lipid peroxidation. Decreased levels of mtDNA have been observed in cardiac tissue from FRDA patients and in FXN deficient yeast models [11]–[13]. The mitochondria appear as the affected organelle in FRDA. ROS powerfully induce peroxisome proliferation activator receptor (PPAR) γ-coactivator 1 (PGC-1α and PGC-1β), that in turn, regulate a complex and multifaceted ROS defense system [14].

In the process of mitochondrial biogenesis, PGC-1α has emerged as a master regulator. PGC-1α is a transcriptional coactivator that transduces many physiological stimuli in important specific metabolic programs, often by stimulating mitochondrial activity [15]. How PGC-1α activates mitochondrial biogenesis has been studied in detail [16]. First, PGC-1α activates nuclear-encoded genes required for mitochondrial biogenesis by co-activating at least three transcription factors termed NRF-1, NRF-2 and ERRα. These transcriptional factors activate certain regulatory promoter regions of several mitochondrial genes encoded in the nuclear genome. PGC-1α also activates the expression of mitochondrial transcription factor A (mtTFA) [16]. Then, mtTFA translocates from the nucleus to the mitochondria, where it stimulates mitochondrial DNA replication and mitochondria gene expression [17], [18]. Furthermore, mtTFA plays a structural role in the maintenance of the mitochondrial chromosome, alternatively of its transcriptional activity. It appears complexed with mtDNA forming nucleoid structures [19] and promoting DNA compaction [20]. It also interacts physically with the protein p53 [21], suggesting a role of mtTFA in DNA repairing mechanisms.

Here, we have characterized the oxidative stress status and mitochondrial biogenesis in FRDA fibroblasts. We observed deficiency of MnSOD, increased ROS levels and low levels of ATP. In addition, we also observed increased expression of PGC-1α and mtTFA and the active form of the upstream signals p38 MAPK and AMPK in two patients. Interestingly, the expression of energetic factors correlated with the natural history of disease of the patients, the age when skin biopsy was performed and the size of the GAA expanded alleles. We propose that induction of mitochondrial biogenesis signals in FRDA could be a consequence of the mitochondrial impairment associated with disease evolution.

## Results

### Evaluation of superoxide and ROS antioxidant enzymes levels in FRDA fibroblasts

First, we tested frataxin expression in fibroblasts from three FRDA by RT-PCR. When expression of *FXN* gene was analyzed by RT-PCR all FRDA patients showed lower mRNA levels than control fibroblasts (Figure S1).

**Figure 1:**
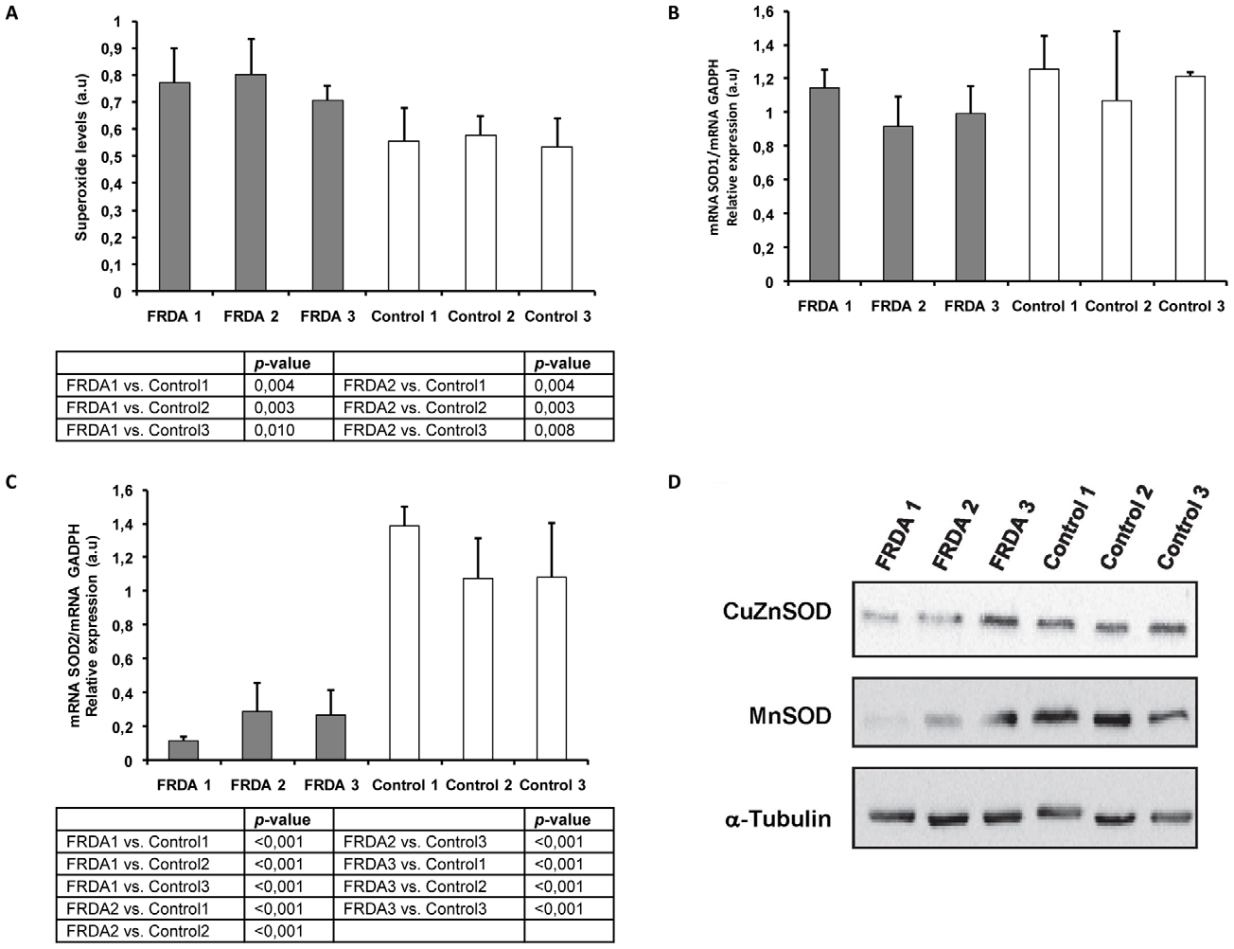
Analysis of the superoxide anion and the superoxide detoxifying enzymes in FRDA and control cells. A. Superoxide levels determined using dihydroethydium probe in FRDA fibroblasts from patients (FRDA 1, FRDA 2, and FRDA 3) and control cells (Control1, Control2, and Control3), results are shown as mean (±SD) of 4–10 different experiments. B. Mean (±SD) mRNA levels of CuZnSOD determined by RT-PCR by triplicate. C. Mean (±SD) mRNA levels of MnSOD determined by RT-PCR by triplicate. D. CuZnSOD and MnSOD protein levels analyzed by western blot in FRDA and control cell lines.

In order to characterize our cellular model (Table 1), we analyzed superoxide levels. We observed significantly higher superoxide anion levels in FRDA patients than in control fibroblasts cell lines, with the exception of the youngest patient (FRDA3) (Figure 1A). Then, we decided to evaluate the antioxidant capacity of FRDA fibroblasts. We measured mRNA and protein levels of several ROS detoxifying enzymes as MnSOD, CuZnSOD, catalase and glutathione peroxidase 1, under basal culture conditions, without inducing oxidative stress. We investigated superoxide metabolism by analyzing the first ROS detoxifying step catalyzed by CuZnSOD and MnSOD, which implies the dismutation of superoxide to hydrogen peroxide and water. We did not observe any significant changes in SOD1 mRNA expression (Figure 1B). However, the CuZnSOD protein levels in FRDA1 and FRDA2 fibroblasts were lower than FRDA 3 or the three control cell lines (Figures 1D). In addition, MnSOD experiments showed a significant reduction of SOD2 mRNA expression (Figure 1C) and protein levels (Figure 1D) of the mitochondrial MnSOD in FRDA 1 and FRDA 2. Our results rather indicate that MnSOD transcription fails in FRDA cultured fibroblasts since mRNA levels appears diminished in FRDA fibroblasts. These data suggest that part of the toxic mechanism in FRDA may involve disruption of the regulatory pathway for MnSOD expression.

**Table 1:** Characterization and identification of FRDA and control cell lines.

Name	Code	GAA repeats	Clinical aspects	Age	sex	Disease onset estimation
**FRDA 1**	GM04078	370/470	Ataxia, mild peripheral neurophathy, cardiomiopathy	30	Male	25
**FRDA 2**	GM03816	350/470	Spinal-cerebral degeneration, cardiomiopathy	36	Female	25
**FRDA 3**	GM03665	780/780	Limb and gait ataxia, scoliosis, proprioceptive sensory loss	13	Female	8
**Control 1**	GM08402	10-10	Apparently healthy subject	32	Male	-
**Control 2**	GM01652	8-8	Apparently healthy subject	11	Female	-
**Control 3**	*	10-10	Apparently healthy subject	50	Female	-

Table shows information of the age, gender and clinical features of individuals whom fibroblasts have been obtained. In addition, the results obtained using genetic analysis of the GAA expanded alleles for the frataxin promoter has been annotated, as well. The procedure for the analysis of GAA triplet expansion was described by Monros et al. [47].

*Control 3 was kindly donated by Dr. Del Rio from the CIEMAT (Madrid, Spain).

**Figure 2:**
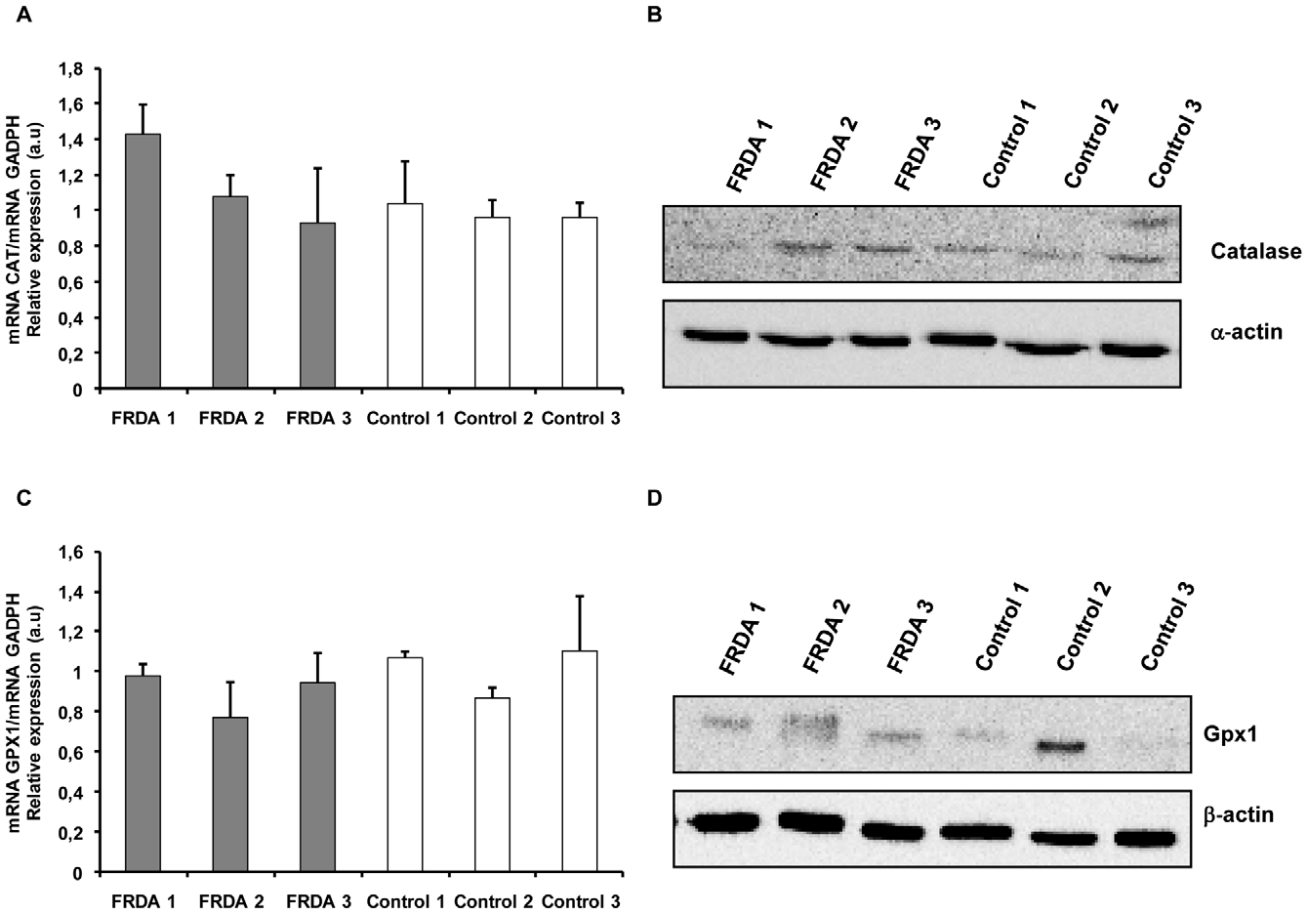
Analysis of hydrogen peroxide detoxifying enzymes in FRDA and control cells. A. Mean (±SD) mRNA levels for CAT gene analyzed by triplicate. B. Catalase protein levels measured by Western blot. C. Mean (±SD) mRNA levels of Gpx1 analyzed by triplicate. D. Determination of Gpx1 protein levels by Western Blot.

In a second step we investigated the status of the peroxide detoxifying enzymes catalase and glutathione peroxidase 1. This detoxification process gains importance in this disease, given that iron accumulation and high levels of H_2_O_2_ can originate Fenton reactions. RT-PCR experiments showed that CAT gene expression was not different between FRDA and control fibroblasts (Figure 2A). In addition, Gpx1 (Figure 2C) mRNA levels were similar in FRDA and control fibroblasts cell lines. Western blot experiments were in accordance with the mRNA expression results for catalase and Gpx1 of the patients' samples (Figure 2B and 2D). The catalase protein levels and mRNA for all three patients showed no differences when compared to their matched control cell lines. In any case, FRDA1 showed low levels for catalase protein compared to the other two FRDA cell lines (Figure 2B). The differences found between CAT expression and catalase protein for FRDA1 may be related to the defects in the correct assembly of the porphyrine heme groups of catalase in these cells.

### Evaluation of SOD, catalase and glutathione peroxidase activities in FRDA fibroblasts

**Figure 3:**
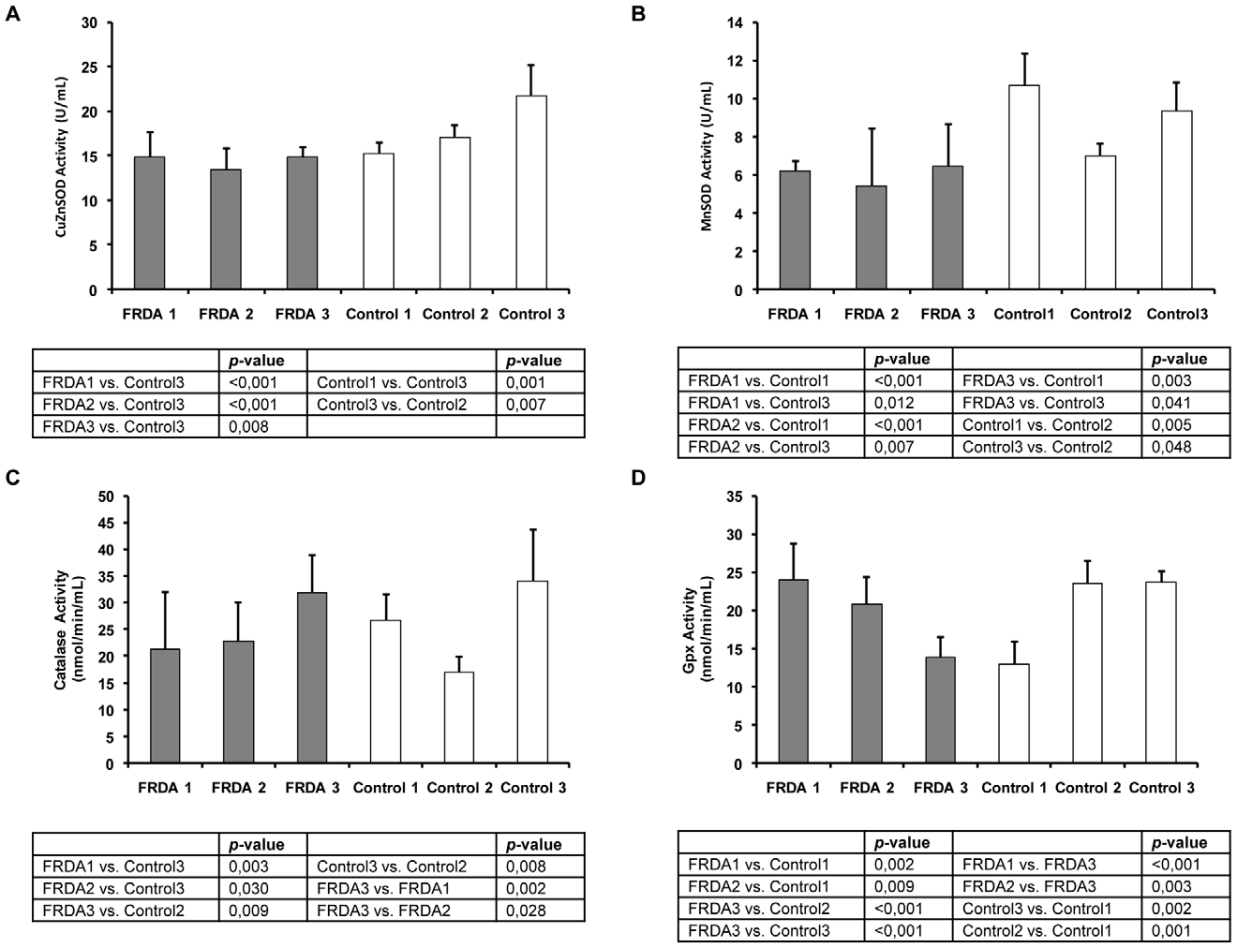
Analysis of anti-oxidant enzymes activities in FRDA and control cells. A. CuZnSOD activity and B. MnSOD activity evaluated using Superoxide dismutase assay kit (Cayman, Ann Arbor, MI, USA) based on the reduction of the tetrazolium salt by the superoxide to give rise formazan that can be measured at 460 nm. Results are represented as Mean (±SD) of 3–4 replicates. C. Catalase activity was analyzed spectrophotometrically measuring at 540 nm the formation of the purpald-formaldehyde adduct. Results are represented as Mean (±SD) of 4–8 replicates. D. Glutathione peroxidase activity determined by the Glutathione peroxidase assay kit (Cayman, Ann Arbor, MI, USA) based on the decrease of NADPH after coupling the glutathione reductase reaction, that use of the NADPH to reduce the GSSG produced by the Gpx after reducing the cumene-hydroperoxide reagent. All measures were made in FRDA fibroblasts from patients (FRDA1, FRDA 2, and FRDA 3) and three control cell lines (Control1, Control2, and Control3). Results are represented as Mean (±SD) of 3–5 replicates.

To further characterize ROS antioxidants enzymes in fibroblasts from FRDA patients we determined the enzymatic activity of the antioxidant systems studied. Superoxide dismutase activities indicated that CuZnSOD activity (Figure 3A) was significantly decreased in FRDA fibroblasts when compared to Control3, one of the adult control cell lines. Results obtained for the mitochondrial isoform of SOD (MnSOD), clearly demonstrated that MnSOD activity was decreased in all FRDA cell lines compared to the activity observed in the two adult control cell lines (Control1 and control3) (Figure 3B). These adult control cell lines showed higher MnSOD activity than fibroblasts obtained from the young control (Control2) as well.

For catalase, results showed in Figure 3C demonstrate that activities were similar in fibroblasts from the FRDA1 patient to their age and gender matched Control1 cell line. However, FRDA1 and FRDA2 fibroblasts showed lower catalase activity than the other adult control cell line (Control3). In contrast, FRDA3 fibroblasts from the youngest patient showed higher catalase activity than their assigned Control2 cell line. Interestingly, FRDA 1 and FRDA2 (fibroblasts from adult patients) showed lower catalase activity than FRDA3 fibroblasts (Figure 3C).

Finally, the studies for glutathione peroxidase activity showed no difference between fibroblasts obtained from FRDA2 patient and their control cells (Control3) (Figure 3D). However, when we compared Gpx activity between FRDA1 fibroblasts with their Control1 we observed higher activity for the former. This was different in the particular case of FRDA3, in which we observed lower Gpx activity than their control cell line (Control2).

### Mitochondrial biogenesis signals are activated in FRDA fibroblasts

As pointed out in the introduction, ROS and low levels of ATP play an important mission as triggers of mitochondrial biogenesis.

**Figure 4:**
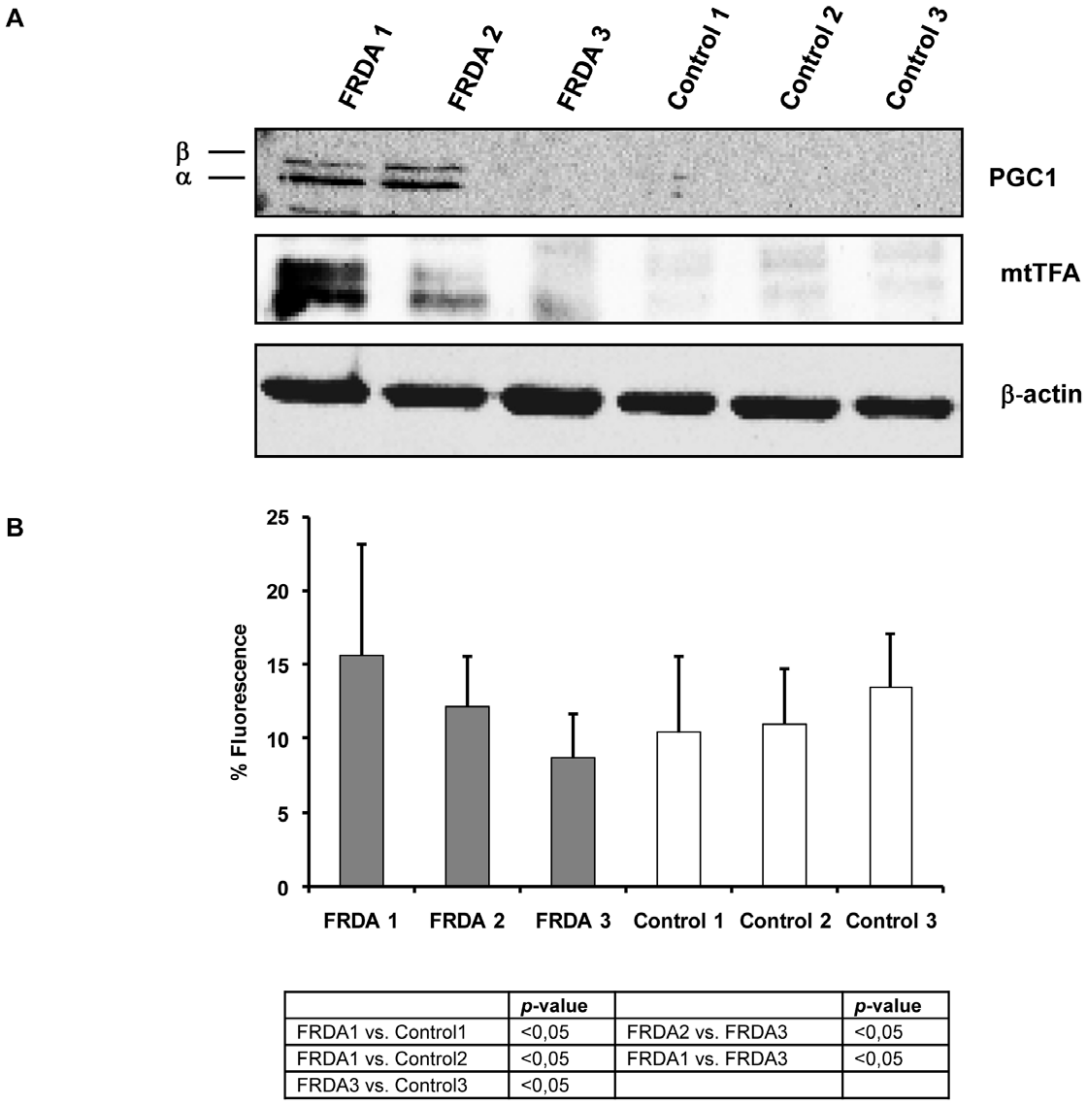
Western blot analysis of peroxisome proliferator-activated receptor co-activator 1α (PGC-1α) in FRDA and control cells. A. Expression of mitochondrial biogenesis-related proteins in FRDA and healthy controls. B. Mitochondrial content in FRDA and healthy controls determined by cytochrome C quantification using confocal microscopy. Results are represented as Mean (±SD) of 180 cells counted at least in 18 different experiments.

We studied the expression of the master regulator of mitochondrial biogenesis PGC-1α and the downstream mitochondrial transcription factor mtTFA. In Figure 4A, we show that relative abundance of PGC-1α and mtTFA was higher in fibroblasts from patients FRDA1 and 2 than in control cells, whereas we did not observe any signal in FRDA3 and in the three control fibroblasts cell lines. These results showed that two different responses for the activation of mitochondrial biogenesis transcription factors can be promoted in fibroblasts deficient for frataxin.

Alteration in intracellular ROS levels has been associated with changes in mitochondrial abundance, mtDNA copy number, and the expression of respiratory genes [22]. Since we found increased levels of PGC-1α and mtTFA, we decided to analyze the mitochondrial content in FRDA and control cells using cytochrome C immunofluorescence detection. Our results, showed in Figure 4B, indicate that FRDA 1 cells are characterized by a higher mitochondrial content than their assigned Control1 and the Control3 cell line. However, FRDA2 cells did not show more cytochrome C levels than control cells. Interestingly, FRDA adult cell lines (FRDA1 and FRDA2) showed higher cytochrome C levels than FRDA 3 fibroblasts (young patient). As we expected, FRDA3 cells did not show an increase in the mitochondrial content.

### Upstream signals involved in the activation of the mitochondrial biogenesis pathway in FRDA fibroblasts

**Figure 5:**
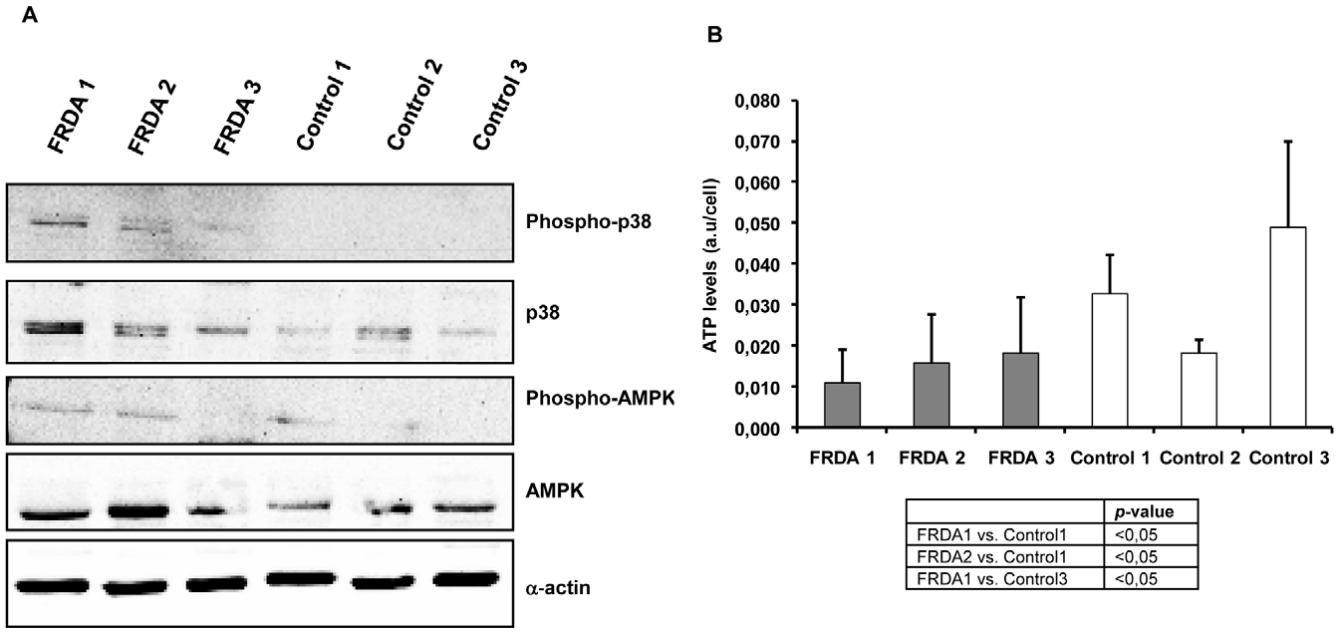
Western blot analysis of upstream proteins involved in PGC-1α overexpression during mitochondrial biogenesis response in FRDA and controls. A. Protein levels of p38 Kinase and phosphorylated p38 and the energy sensor AMPKα and phospho-AMPKα. B. ATP levels measured by luciferase assay. Results are represented as Mean (±SD) of at least six independent experiments.

We also studied the upstream activation pathways of PGC-1α in order to provide insight into the cellular mechanism by which mitochondrial biogenesis transcription factors were increased in FRDA cells from the two adult patients (FRDA1 and FRDA2). We observed the activation of p38 MAPK due to phosphorylation of Thr180/Tyr182 in these two FRDA patients (Figure 5A).

When we analyzed cellular levels of the energy status sensor, the AMP activated kinase (AMPK), we observed increased activated AMPK levels (phosphorylated form) in FRDA 1 and FRDA 2, but not in the FRDA 3 or control cells, suggesting the implication of AMPK in the mitochondrial biogenesis activation process (Figure 5A). This kinase is activated by an increase in the intracellular AMP/ATP ratio, for this reason is considered as a cellular energy sensor. In order to corroborate these results, we decided to analyze ATP levels in the different cell lines. The analysis of ATP levels by enzymatic assay using luciferase showed that ATP content was lower in FRDA 1 and FRDA 2 fibroblasts than in their matched controls (Control1 and Control3, respectively) (Figure 5B).

### Antioxidant idebenone modulates mitochondrial biogenesis response

As we have mentioned, ROS could stimulate mitochondrial biogenesis. This idea was postulated almost thirty years ago [23]. Furthermore, we have described how the antioxidant vitamin C decreases mitochondriogenic responses during exercise [24]. For this reason we decided to check the cellular mitochondrial biogenesis signals after idebenone treatment.

Idebenone, an antioxidant used as therapeutic agent in FRDA has shown to delay the cardiac disease onset, demonstrating that it is cardioprotective, even when there is a complete lack of frataxin [25].

**Figure 6:**
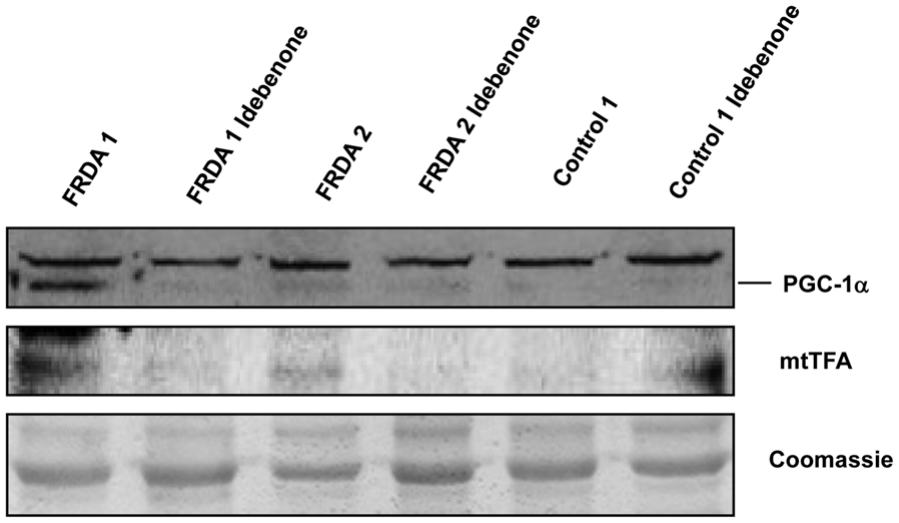
Western blot of PGC-1α and mtTFA after treatment with idebenone. Samples were obtained from FRDA 1 and 2 and Control1 fibroblasts incubated with 5 µM of Idebenone during 5 days in 5% CO_2_ in air at 37°C at density of 20,000 cells/cm^2^.

In view of the previous results, we decide to analyze if idebenone administration could affect mitochondrial biogenesis responses. Results observed in Figure 6 show that the mitochondrial biogenesis pathway was inhibited by idebenone. Both, PGC1α and mtTFA decreased in FRDA1 and FRDA2 after fibroblasts incubation with cell culture medium containing idebenone, underscoring the role of ROS and the efficiency of the mitochondrial electron transfer reactions in the induction of PGC1α and mtTFA in FRDA fibroblasts.

## Discussion

Friedreich's ataxia is a mitochondrial rare disease, which molecular origin is associated with a defect in the expression of frataxin. The pathological consequences are degeneration of nervous system structures, mainly sensory neuropathy in dorsal root ganglia, accompanied by degeneration of posterior columns in the spinal cord and pyramidal tracts, and cardiomyopathy with necrosis and fibrosis. Oxidative stress as a relevant pathogenic mechanism in FRDA has been well established. By contrast, only recently mitochondrial biogenesis related transcriptional factor PGC-1α has been studied in FRDA [26]. The pathological process is progressive and chronic and evolving differences over time may be relevant to understand the biochemical and cellular changes conditioning the pathophysiology of the disease.

We detected abnormal parameters associated with energetic unbalance and oxidative stress in fibroblasts from the three patients: ATP levels were reduced, superoxide anion was increased and SOD expression was significantly reduced. These data agree with previous reports in different cell types [27], [28]. Previous studies have shown that up-regulation of MnSOD fails to occur in FRDA fibroblasts exposed to iron [28], [29] or by oligomycin-induced oxidative stress [28]. Cardiac Frda/MCK conditional mouse models lacking frataxin, that reproduce the biochemical and pathophysiological features of the human disease, showed decreased levels in MnSOD transcript and protein in the late stages of the disease [25].

Mitochondria are the most important subcellular site of O_2_^•−^ and H_2_O_2_ production in mammalian cells. The steady state concentration of O_2_^•−^ in the mitochondrial matrix is about 5- to 10-fold higher than that in the cytosolic and nuclear spaces. Hence, mitochondrial components and mtDNA can be damaged by O_2_^•−^ and H_2_O_2_ [30]. ROS are part of the mitochondria-nucleus signaling pathway, and can mediate some responses to stimulate mitochondrial biogenesis signals. These responses have been described in HeLa cells in which, mtDNA had been depleted by ROS [31]. In our case, the FRDA1 and FRDA2 fibroblasts lines from FRDA patients showed higher levels of superoxide radical than control fibroblasts or FRDA3 cell line (Figure 1A; Table S1). We consider that FRDA 1 and FRDA 2 fibroblasts are more predisposed to oxidative stress because they have less CuZnSOD and MnSOD protein levels than FRDA 3 and control fibroblasts (Figure 1D). Furthermore, CuZnSOD activities were lower in FRDA fibroblasts than the adult control cell line, Control3 (Figure 3A; Table S2). The MnSOD activity in FRDA cell lines was also lower than in adult controls (Control1 and Control3) (Figure 3B). Thus, the low levels of protein and activities of both SOD enzymes in FRDA1 and FRDA2 fibroblasts (mainly for FRDA1) give cells lower antioxidant defence, and therefore should be more predisposed to oxidative stress (Figure 1A; Table S2). This is an interesting point since, FRDA 3 showed the longest GAA triplet expansion (Table 1) and the lowest levels of mRNA FXN levels of the three patients analyzed (Figure S1; Table S1), suggesting, that SOD depletion may be related with the progression of the disease.

By contrast, we did not observe significant differences in other antioxidant enzymes. The catalase mRNA and protein levels (Figure 2A and 2B; Table S1) were similar between FRDA and control fibroblasts. The study of the catalase activity showed a particular pattern for FRDA and control fibroblasts (Figure 3C; Table S2). Catalase activity for FRDA1 was similar to Control1 fibroblasts (Figure 3C, Table S2). However, catalase activity for FRDA2 was lower than their matched Control3, while catalase activity for FRDA3 was higher than Control2. These differences may be because oxidative stress conditions have not been induced in our cell culture model, to increase the antioxidant responses, as was carried out in a previous report by Chantre-Groussard et al [28]. Interestingly, catalase activity in FRDA1 and FRDA2 were lower than in FRDA3 cells, suggesting that antioxidant enzymes unbalance may be related with the progression of the disease.

Furthermore, our results did not show any differences in glutathione peroxidase 1 expression (Figure 2C) when we compared control and FRDA fibroblasts. These results were similar to those obtained by Jauslin et al. [32]. However, when we analyzed total glutathione peroxidase activity, we only observed differences in Gpx total activity for FRDA1 compared to their matched control (Figure 3D; Table S2). Probably, the high Gpx activity in FRDA1 and FRDA2 cell lines compared with FRDA3 is a cellular effort to compensate the low levels of catalase.

Under our opinion, these studies indicate that the levels of CuZnSOD and the MnSOD are lower *per se* in all FRDA patients, (and more drastically in FRDA 1 and FRDA 2 cells) suggesting that, the mitochondrial pathogenesis of FRDA could be aggravated by deficit in these variants of superoxide dismutase and that the critical evolution of the disease with the age of patients is accompanied by the depletion of the MnSOD.

The low levels of MnSOD and ATP (Figure 1D and Figure 5B, respectively) measured in FRDA 1 and 2 cells indicate the involvement of mitochondria in the physiopathology of Friedreich's ataxia, as it has been described by other authors [33]. For this reason is necessary to elucidate distinct signaling pathways that may occur in the cell to restore mitochondria function.

Oxidative stress has been involved in the activation of mitochondrial biogenesis responses, through activation of different signaling pathways [31], [34], [35].

There is evidence that oxidative stress and mtDNA damage enhance the expression of nuclear mitochondrial biogenesis genes, and ROS constitute a part of a mitochondria-nucleus regulatory signaling pathway [31], [35]. Furthermore, Suzuki et al., have described that the expression of mitochondrial biogenesis is governed by genes such as, mtTFA and NRF-1; and their DNA-binding activities were increased in human cells with impaired respiratory function [34].

The analysis of protein levels of PGC-1α and mtTFA proteins in FRDA cells indicate an over-expression for both proteins in FRDA 1 and FRDA 2 fibroblasts (Figure 4A), but not in FRDA 3 cells or control cells. The mitochondrial content in FRDA cell lines analyzed by cytochrome C immunofluorescence confirmed that only FRDA1 fibroblasts contained more mitochondria (Figure 4B; Table S3). These results were consistent with the downstream signal linked to mtTFA expression, in which mtTFA levels were lower in FRDA2 than in FRDA1 fibroblasts (Figure 4A). Although the whole upstream signaling involved in the activation of PGC-1α is yet to be determined, several pathways have been described. At the moment some mechanisms have been clarified, and the activity of PGC-1α can be modulated by numerous post-translational events, including phosphorylation of the p38 MAPK [36], [37] and AMP kinase (AMPK) [38], among others. We have observed up-regulation of the phosphorylated active form of both p38 MAPK and AMPK kinases in fibroblasts from FRDA 1 and FRDA 2 patients but not in FRDA 3 cells or control cell lines, indicating that it is a feasible phenomenon involved in the activation of PGC-1α (Figure 5A). The observed activation of these pathways in FRDA1 and FRDA2 cells might be originated by the increased levels of ROS, p38 MAPK and reduction of ATP levels (Figure 5B; Table S1; Table S3) and the subsequent activation of AMPK pathway (Figure 5) [39]–[41].

A recent publication by Coppola et al. [42] has shown different expression pattern of PGC-1α in tissues from frataxin-deficient mice. In that case, they showed low levels of PGC-1α in several tissues with the notable exception of cardiac muscle. This exception is remarkable because it has been demonstrated that continuous expression of PGC-1α in the myocardium, resulted in a dilated cardiomyopathy [43]. Coppola et al. studied fibroblast obtained from skin biopsies as well. In skin fibroblasts they observed that PGC-1α was down-regulated in FRDA. Although apparently their results are different to those obtained by us (Figure 4), an analysis of their experimental procedure indicates that fibroblasts were obtained from skin biopsies of FRDA patients treated with idebenone (5 mg/kg).

In order to explain our results we try to identify if idebenone, an antioxidant used as a therapeutic agent in FRDA, could have inhibitory effects on the mitochondrial biogenesis response. Previous works in other physiological situations using animal models have shown that the administration of vitamin C significantly decreases the expression of transcription factors involved in mitochondrial biogenesis [24].

Coenzyme Q_10_ and its analog idebenone are being used for the treatment of FRDA. CoQ_10_ increase the efficiency of electron transfer through the respiratory chain and acts as an antioxidant itself [44]. The effect of CoQ_10_ or the analog idebenone upon cardiac hypertrophy in FRDA patients has been assessed using echocardiography. After 6 months treatment with idebenone, cardiac hypertrophy was decreased in up to half the patients tested, although this was not always associated with improved fraction shortening [45].

We propose that fibroblasts in absence of antioxidants have higher levels of PGC-1α. However, when idebenone is added to cells produces the inhibition of mitochondrial biogenesis responses (Figure 6) by affecting, potentially, two pathways: Idebenone may reduce ROS levels into the cells, reducing p38 activation and, idebenone may improve the efficiency of the mitochondrial respiratory chain, and then decreasing the levels of activated AMPK. Our results are in accordance with the observations showing that treatment with idebenone improves cardiac hypertrophy [45] and could represent a feasible explanation to the mechanism mediated by idebenone to improve the cardiac outcomes.

Thus, PGC-1α appears as a downstream effector of frataxin deficiency. Moreover, idebenone may affect the downstream signals, improving the pathophysiological consequences of frataxin deficiency.

We have investigated the oxidative status and mitochondrial bioenergetic regulation in fibroblasts from three FRDA patients. We detected abnormal oxidative status and reduction of ATP in fibroblasts from all of them; by contrast, the molecular analysis of mitochondrial biogenesis signals showed two different patterns: while FRDA1 and FRDA2 samples showed increase expression of mtTFA transcription factor and the master regulator of mitochondrial biogenesis PGC-1α, and also of the active phosphorylated forms of p38 MAPK and the metabolic sensor AMPK. No metabolic response was detected in fibroblasts from patient FRDA3. Thus, there was no full coincidence on the oxidative status and biogenesis metabolism in mitochondria.

There are, however, some clinical and genetic differences among the patients that could explain the observed differential status of oxidative stress and biogenesis response. Patients FRDA1 and 2 were 30 and 36 years old, respectively, when skin biopsy was taken. Both individuals were carrying one GAA expanded allele with a number of repeats under the threshold of 500 that is usually associated with a late onset of the disease (Table 1) [3], [46]. Both patients showed cardiomyopathy, as well (Table 1). Based on the GAA expansion of the smaller allele [46] the age at onset in both patients FRDA 1 and FRDA 2 could be estimated at 25 years (Table 1). In the same way the age at onset of patient FRDA 3 could be estimated at 8 years (Table 1); when skin biopsy was performed at the age of 13 no cardiomyopathy was referred to (Table 1). We propose that increase of ROS and involvement of the oxidative phosphorylation may be an early event in the cell pathophysiology of frataxin deficiency, whereas increase of mitochondriogenic response might be a later phenomenon associated to the individual age and natural history of the disease, being more evident as the patient age increases and disease evolves. This is a possible explanation of heart disease in FRDA. Several studies have suggested a correlation between cardiomyopathy and overexpression of PGC-1 [43], [47]. In that way, mitochondrial proliferation, a well recognized compensatory mechanism in mitochondrial disease, could contribute to myocardium remodeling because mitochondrial proliferation could interfere with sarcomere alignment and contraction [47]. Although more studies are necessary to translate these observations to cells typically affected in FRDA, such as neurons and cardiomyocytes, our observations in fibroblasts suggest a role of mitochondrial biogenesis signals in the clinical evolution of the Friedreich's ataxia pathophysiology. Due to the rarity of the disease, the scarcity of samples, the involved nature of the analyses, and the interindividual variability of both the patients and the healthy controls, it is difficult to establish firmly the role of PGC-1α and other metabolic sensors on mitochondrial biogenesis in Friedreich's ataxia. But it is becoming increasingly evident from this and other studies that the role of oxidative stress and mitochondria must be taken into account if we want to build up a *complete picture* of the pathophysiology of Friedreich's ataxia.

## Materials and Methods

### Cell Culture

FRDA fibroblasts (GM04078, GM03816 and GM03665) and control fibroblasts (GM08402 and GM01652) were obtained from Coriell Cell Repository (Camden, NJ). Control fibroblasts are age and gender matched, being GM04072 the FRDA1 (male, 30 years); GM03816 the FRDA2 (female, 36 years); GM03665 the FRDA3 (female, 13 years); GM08402 the Control1 (male, 32 years) and GM01652 the Control2 (female, 11 years). An additional female adult control (Control3) was kindly donated by Dra. Del Rio from the CIEMAT (Madrid, Spain). The cells were cultured in Eagle's minimum essential medium with Earle's salts and non-essential amino acids (DMEM, Gibco, Invitrogen) supplemented with 15% fetal bovine serum inactivated, 1% Glutamine and 1% penicillin-streptomycin (Sigma-Aldrich, St. Louis, MO) in 5% CO_2_ in air at 37°C at density of 20,000 cells/cm^2^. Trypsin-EDTA was used as the subculture method. Studies were performed at cell confluence. Characteristics and clinical aspects of patients as reported at the Coriell website have been collected in Table 1. Genetic analysis of the GAA expanded alleles were performed as previously reported by Monrós et al. [47] and have been collected in Table 1, as well. In some experiments, FRDA and control fibroblasts were cultured in presence of idebenone. Briefly, cells were incubated with idebenone (5 µM) during 5 days using experimental conditions reported previously by Jauslin et al. [48].

### Superoxide levels determination

Cells were cultured in 6 chamber plates for 6 days (at confluence). After cells were washed 2 times with pre-warmed PBS medium, 2 µL/mL of diluted dihydroethidium (Sigma, St. Louis, USA) was added to the plate. Cells were incubated at 37°C for 20 min. After washing the plate with PBS, medium was replaced for another one without dihydroethidium for 1 hour at 37°C. The fluorescence was measured using fluorimeter spectraMAX GEMINIS (Molecular Devices, Sunnyvale, USA), with 530 nm of excitation wavelength and 610 nm of emission wavelength. All samples were analyzed between 4–10 independent experiments.

### Real-time quantitative PCR

#### RNA isolation and cDNA synthesis

Total RNA was isolated from cells using the PARIS™ Protein and RNA Isolation System (Ambion, Austin, TX) according to the manufacturer's instructions. For reverse transcription reactions (RT), 1 µg of the purified RNA was reverse transcribed using random hexamers with the High-Capacity cDNA Archive kit (Applied Biosystems, P/N: 4322171; Foster City, CA) according to the manufacturer's instructions. RT conditions comprised an initial incubation step at 25°C for 10 min. to allow random hexamers annealing, followed by cDNA synthesis at 37°C for 120 min, and a final inactivation step for 5 min. at 95°C.

#### Measurement of mRNA Levels

The mRNA levels were determined by quantitative real-time PCR analysis using an ABI Prism 7900 HT Fast Real-Time PCR System (Applied Biosystems, Foster City, CA). Gene-specific primer pairs and probes for *FXN (Frataxin)*, *SOD1* (*SOD Cu/Zn*), *SOD2* (*SOD Mn*), *GPX1 (Glutathione peroxidase 1)* and *CAT* (*Catalase*) (Assay-on-demand, Applied Biosystems), were used together with 1× TaqMan® Universal PCR Master Mix (Applied Biosystems, P/N 4304437, Foster City, CA) and 2 µl of reverse transcribed sample RNA in 20 µl reaction volumes. PCR conditions were 10 min. at 95°C for enzyme activation, followed by 40 two-step cycles (15 sec at 95°C; 1 min at 60°C). The levels of glyceraldehyde-3-phosphate dehydrogenase (*GAPDH*) expression were measured in all samples to normalize gene expression for sample-to-sample differences in RNA input, RNA quality and reverse transcription efficiency. Each sample was analyzed in triplicate, and the expression was calculated according to the 2^−ΔΔCt^ method [49].

### Cell lysates and Western blot analysis

Approximately 3·10^6^ cells were lysed using lysis buffer (Hepes, pH 7.4, 20 mM, tritonX-100 1%, NaCl 100 mM, NaF 50 mM, β-glycerophosphate 10 mM, activated sodium orthovanadate 1 mM, PMSF 1 mM, protein proteases inhibitor cocktail 2 µL/mL in ice about 15 minutes and then the suspension was spun-down at 13000 g for 10 min at 4°C and the supernatants were collected and stored at −80°C until their use. Protein content was determined by a modified Lowry method [50]. Aliquots of cell lysates (40–50 µg) were added to sample buffer with 10% β-mercaptoethanol and then were immediately boiled for 5 min and separated by electrophoresis in sodium dodecyl sulfate 12% polyacrylamide gels (SDS-PAGE), 100V during two hours. After electrophoresis, the proteins were electroblotted (Bio-Rad) onto nitrocellulose membrane. Membranes were blocked with 0.05 g/ml non-fat milk or BSA 0.05 g/ml in TBS-0.2% Tween 20 (TBST) according to the antibody, washed three times at room temperature, and incubated with primary antibodies against catalase (1∶1000) (Sigma, St. Louis, USA), MnSOD (1∶1000) (Stressgen, Ann Arbor, MI, USA), CuZnSOD (1∶1000, Stressgen, Ann Arbor, MI, USA), Gpx1 (1∶750, Abcam, Cambridge, MA, USA), PGC1 (1∶500, Cayman Chem. Ann Arbor, MI, USA), PGC1 (1∶750, Santa Cruz BioTech. USA), mtTFA (Santa Cruz BioTech. California, USA), p38 and phosphorylated-p38 (1∶1000, Cell Signaling, Boston, MA, USA), AMPK and phosphorylated-AMPK (1∶1000, Cell Signaling, Boston, MA, USA) and α-tubulin or α-actin (1∶1000, Santa Cruz BioTech. USA) as loading control, in TBST with 0.01 g/ml non-fat milk for 2 h at room temperature. Thereafter, the blots were washed again with TBST and further incubated for 1 h with a secondary mouse, rabbit or goat antibody conjugated with horseradish peroxidase-linked. After washing with TBST as above, blots were developed by using the ECL™ Western Blotting Detection Reagents as specified by the manufacturer (Amersham GE HealthcareBio-Sciences AB, Uppsala, Sweden). Chemioluminescent signals were assessed using a Fujifilm scanning densitometer (Fujifilm LAS-1000 plus).

### Measurement of CuZnSOD and MnSOD activity

To determine MnSOD and CuZnSOD activity the cells were treated as is described in the Cayman “Superoxide Dismutase Assay kit” (Ann Arbor, MI). After centrifugation at 10,000 g for 10 min, supernatant was used to measure CuZnSOD activity. The mitochondrial pellet was lysed using a lysis buffer compatible with the manufacturer's instructions (10 mM HEPES, pH7.9, 420 mM NaCl, 1,5 mM MgCl_2_, 0,5 mM EDTA, 0.1% Triton X-100) for 20 min on ice. After centrifugation at 12,000 g for 5 min, the supernatant was collected for MnSOD activity assay. Measurements of CuZnSOD and MnSOD activities were performed in a 96 well plate prepared using 3–4 replicates from different cellular extracts for each sample. The final absorbance was measured at 450 nm using a spectrophotometer spectraMAXPLUS 384 (Molecular Devices, Sunnyvale, CA, USA).

### Measurement of catalase activity

The method for measuring the catalase enzymatic activity was based on the reaction of the enzyme with methanol in the presence of hydrogen peroxide to produce formaldehyde. Cells were lysate using freeze (liquid N_2_, 10 s) and thaw (ice, 15 min) procedure repeated three times. After centrifugation of the cell lysate at 13000 rcf, for 10 min. at 4°C, supernatants were recovered and quantified using Lowry method. A 96 well plate was prepared using at least 4 replicates for each sample, obtained from different cellular extracts.

Assay reaction consisted in mixing on a 96 well plate: 100 µL of Kpi 100 mM pH 7.0; 30 µL methanol and 20 µL of the sample with the same protein concentration). Then, the reaction was started with 20 µL of 85 mM H_2_O_2_, maintained during 20 min at room temperature and finally stopped using 30 µL of KOH 10 M. The formaldehyde produced reacts with 35 mM purpald reagent dissolved in 0,5 M HCl during 10 min at room temperature. Finally, 10 µL of 0.5% KIO_4_ in KOH 0.5 M were added and the absorbance at the wavelength of 540 nm was measured with spectrophotometer spectra MAXPLUS 384 (Molecular Devices, Sunnyvale, CA, USA).

### Measurement of glutathione peroxidase activity

Gpx activity was measured by using a glutathione peroxidase assay kit (Cayman (Ann Arbor, MI). Briefly, cells were collected and lysated using cold buffer (50 mM Tris-HCl, pH 7.5, 5 mM EDTA and 1 mM DTT) and two freeze-thaw cycles with liquid N_2_. The lysates were centrifuged at 10000 g for 15 min at 4°C and the supernatants recovered in fresh tubes. A 96 well plate was prepared using at least 3 replicates for each sample from different cellular extracts. After protein quantification by Lowry method, samples containing 20 µg of total proteins were added to the 96 well plate containing a solution with 1 mM GSH, 0.4 U/mL of glutathione reductase, 0.2 mM NADPH. The reaction was initiated by adding 0.22 mM of cumene hydroperoxide and the reduction of the absorbance was recorded at 340 nm each 1 min during 8 min. The Gpx activity was determined by the rate of decrease in absorbance at 340 nm (1 mU/mL Gpx). Molar coefficient extinction for NADPH was 0.00622 mM^−1^ cm^−1^, and the pathlength of the solution into the plate was established in 0.6 cm.

### Fluorescent microscopy to evaluate mitochondrial distribution and morphology

Cells were grown on coverslips inside a petri dish filled with DMEM. 24 h later cells were fixed with 2% and 4% solution paraformaldehyde successively and then permeabilized with a solution of 0.5%PBS-Triton X-100, at 37°C, 5% CO_2_ during 10 min. Cells were probed with anti-cytochrome-C antibody (Zymed, Millipore, Billerica. USA) in blocking solution (PBS/FBS 3%) and then incubated with fluorescent Alexa Fluor 488 secondary antibody (Molecular probes). Appropriate negative controls were made by incubating fixed cells with secondary antibodies only. Coverslips were then fixed on microscope slides and digitized with a Hamamatsu camera (Tokyo, Japan) connected to Leica DMR microscopy (Nussloch, Germany). All images were captured under constant exposure time, gain, and offset. Fluorescence measurements were made using ImageJ and relative to the cell surface. At least 180 cells were counted for each cell type in at least 18 different experiments and the mean ± SD calculated.

### Quantification of ATP levels

Briefly, cells were trypsinized and resuspended in 0.5 mL PBS (1×10^6^ cells/mL). The ATP levels were determined using the adenosine 5′-triphosphate (ATP) Bioluminiscent Assay (Sigma, St. Louis, USA) following the manufacturer's instructions After releasing of the ATP from the cells, bioluminescent signal was measured in triplicate for each cell type using Wallac Victor2TM 1420 multilabel Counter (PerkinElmer, Waltham, MA, USA) in at least six independent experiments. Results were represented as mean ± SD.

### Statistical analyses

For the statistical analysis of the results, the mean was taken as the measurement of the main tendency, while standard deviation was taken as the dispersion measurement. A one way analysis of variance was used to determine the difference between groups analyzing superoxide levels, expression of FXN, SOD1, SOD2, CAT and GPX1 genes and activities for MnSOD, CuZnSOD, catalase and glutathione peroxidase. When an interaction effect was found, multiple comparisons using the Student-Newman-Keuls method post hoc test were performed. When the normality test failed (ATP and cytochrome C values) we performed a Kruskal-Wallis one way ANOVA on ranks with multiple comparisons using Dunn's method. Each measure was performed using independent experiments. Different number of technical replicates was used in each analytical determination (see specific technique for details).

The alpha level for statistical significance was set at p<0.05.

## Supporting Information

Figure S1 **Frataxin levels determined in FRDA and control cells.** Mean (±SD) mRNA levels of frataxin in FRDA (FRDA1, FRDA2, and FRDA3) and control cells (Control1, Control2, Control3) analyzed by triplicate. Results show lower frataxin levels in FRDA than in control fibroblasts.
(TIF)

Table S1 **Experimental values obtained for superoxide and mRNA levels for distinct antioxidant enzymes.** Table shows all experimental data as Mean (±SD) obtained for superoxide quantification; mRNA levels for FXN, SOD1, SOD2, CAT and GPX1 genes determined by RT-PCR.
(DOCX)

Table S2 **Experimental values obtained for antioxidant enzymatic activities.** Table shows all experimental data as Mean (±SD) obtained for CuZnSOD, MnSOD, catalase and total glutathione peroxidase activity.
(DOCX)

Table S3 **Experimental values obtained for Cytochrome C and ATP quantification.** Table shows all experimental data as Mean (±SD) obtained for Cytochrome C levels quantified using immunofluorescence and ATP levels quantified using the luciferase assay.
(DOCX)
