# Supplementary figures and images for: Differential Expression of PGC-1α and Metabolic Sensors Suggest Age-Dependent Induction of Mitochondrial Biogenesis in Friedreich Ataxia Fibroblasts
Source: PLoS One. 2011 Jun 7;6(6):e20666. doi: 10.1371/journal.pone.0020666 (PMC3110204; doi:10.1371/journal.pone.0020666)

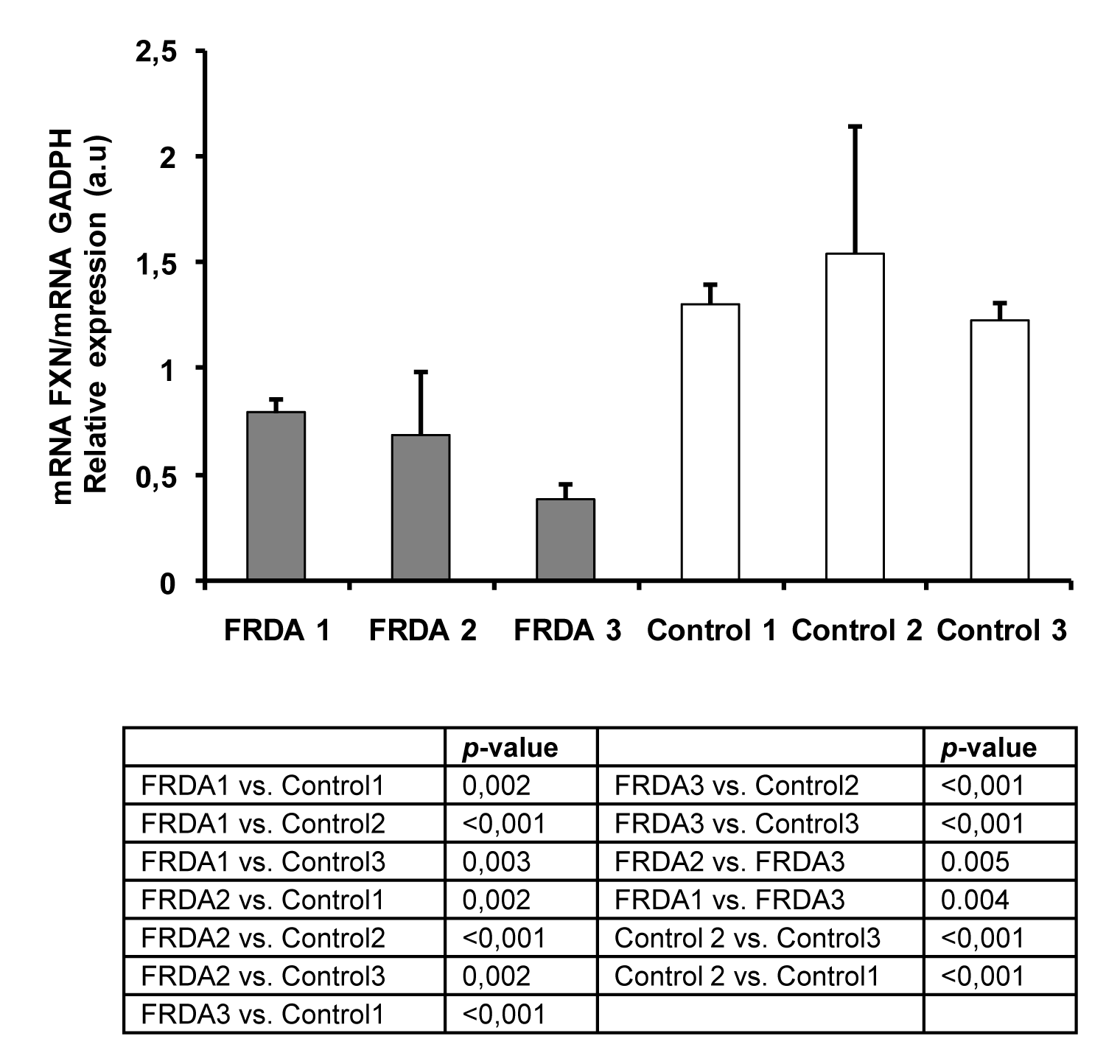

Supplement: Figure S1 — Frataxin levels determined in FRDA and control cells. Mean (±SD) mRNA levels of frataxin in FRDA (FRDA1, FRDA2, and FRDA3) and control cells (Control1, Control2, Control3) analyzed by triplicate. Results show lower frataxin levels in FRDA than in control fibroblasts. (TIF) [file pone.0020666.s001.tif]
