# Supplementary material for: Differential Expression of PGC-1α and Metabolic Sensors Suggest Age-Dependent Induction of Mitochondrial Biogenesis in Friedreich Ataxia Fibroblasts
Source: PLoS One. 2011 Jun 7;6(6):e20666. doi: 10.1371/journal.pone.0020666 (PMC3110204; doi:10.1371/journal.pone.0020666)
Supplement: Table S1 — Experimental values obtained for superoxide and mRNA levels for distinct antioxidant enzymes. Table shows all experimental data as Mean (±SD) obtained for superoxide quantification; mRNA levels for FXN, SOD1, SOD2, CAT and GPX1 genes determined by RT-PCR. (DOCX) [file pone.0020666.s002.docx]

**Table S1. Experimental values obtained for superoxide and mRNA levels for distinct antioxidant enzymes.** Table shows all experimental data as Mean (±SD) obtained for superoxide quantification; mRNA levels for FXN, SOD1, SOD2, CAT and GPX1 genes determined by RT-PCR.

|  | **O_2_^·-^**  **(a.u)** | **mRNA**  **SOD1**  **(a.u)** | **mRNA**  **SOD2**  **(a.u)** | **mRNA**  **CAT**  **(a.u)** | **mRNA**  **GPX1**  **(a.u)** | **mRNA**  **FXN**  **(a.u)** |
| --- | --- | --- | --- | --- | --- | --- |
| **FRDA 1** | 0,77±0,12 | 1,14±0,10 | 0,12±0,027 | 1,42±0,16 | 0,98±0,05 | 0,77±0,04 |
| **FRDA 2** | 0,80±0,11 | 0,91±0,18 | 0,28±0,16 | 1,07±0,12 | 0,77±0,17 | 0,80±0,22 |
| **FRDA 3** | 0,70±0,05 | 0,99±0,14 | 0,23±0,09 | 0,93±0,31 | 0,94±0,14 | 0,37±0,05 |
| **CONTROL 1** | 0,55±0,12 | 1,25±0,19 | 1,36±0,09 | 1,04±0,23 | 1,07±0,02 | 1,30±0,09 |
| **CONTROL 2** | 0,58±0,07 | 1,06±0,41 | 1,13±0,19 | 0,96±0,09 | 0,86±0,05 | 1,87±0,23 |
| **CONTROL 3** | 0,54±0,10 | 1,21±0,02 | 1,16±0,26 | 0,96±0,08 | 1,10±0,27 | 1,23±0,07 |
