# Supplementary material for: Differential Expression of PGC-1α and Metabolic Sensors Suggest Age-Dependent Induction of Mitochondrial Biogenesis in Friedreich Ataxia Fibroblasts
Source: PLoS One. 2011 Jun 7;6(6):e20666. doi: 10.1371/journal.pone.0020666 (PMC3110204; doi:10.1371/journal.pone.0020666)
Supplement: Table S2 — Experimental values obtained for antioxidant enzymatic activities. Table shows all experimental data as Mean (±SD) obtained for CuZnSOD, MnSOD, catalase and total glutathione peroxidase activity. (DOCX) [file pone.0020666.s003.docx]

**Table S2. Experimental values obtained for antioxidant enzymatic activities.** Table shows all experimental data as Mean (±SD) obtained for CuZnSOD, MnSOD, catalase and total glutathione peroxidase activity.

|  | **Activity**  **CuZnSOD**  **(U/mL)** | **Activity**  **MnSOD**  **(U/mL)** | **Activity**  **Catalase**  **(nmol/min/mL)** | **Activity**  **Gpx**  **(nmol/min/mL)** |
| --- | --- | --- | --- | --- |
| **FRDA 1** | 14,04±1,54 | 5,19±0,89 | 19,09±5,91 | 23,93±4,87 |
| **FRDA 2** | 13,48±2,32 | 4,93±2,45 | 22,76±7,37 | 20,73±3,59 |
| **FRDA 3** | 14,86±1,08 | 6,46±1,56 | 31,95±6,88 | 13,83±2,71 |
| **CONTROL 1** | 15,17±1,29 | 12,13±1,49 | 26,70±4,89 | 12,83±3,04 |
| **CONTROL 2** | 17,12±1,34 | 7,18±0,58 | 17,04±2,84 | 23,50±2,97 |
| **CONTROL 3** | 21,73±3,46 | 9,96±0,99 | 34,09±9,66 | 23,69±1,43 |
