# Supplementary material for: Differential Expression of PGC-1α and Metabolic Sensors Suggest Age-Dependent Induction of Mitochondrial Biogenesis in Friedreich Ataxia Fibroblasts
Source: PLoS One. 2011 Jun 7;6(6):e20666. doi: 10.1371/journal.pone.0020666 (PMC3110204; doi:10.1371/journal.pone.0020666)
Supplement: Table S3 — Experimental values obtained for Cytochrome C and ATP quantification. Table shows all experimental data as Mean (±SD) obtained for Cytochrome C levels quantified using immunofluorescence and ATP levels quantified using the luciferase assay. (DOCX) [file pone.0020666.s004.docx]

**Table S3. Experimental values obtained for Cytochrome C and ATP quantification.** Table shows all experimental data as Mean (±SD) obtained for Cytochrome C levels quantified using immunofluorescence and ATP levels quantified using the luciferase assay.

|  | **Cytochrome C**  **% Fluoresc (a.u)** | **ATP levels**  **(a.u/cell)** |
| --- | --- | --- |
| **FRDA 1** | 15,66±7,43 | 0,011±0,008 |
| **FRDA 2** | 12,13±3,38 | 0,016±0,012 |
| **FRDA 3** | 8,65±3,04 | 0,018±0,013 |
| **CONTROL 1** | 10,39±5,13 | 0,032±0,009 |
| **CONTROL 2** | 11,02±3,63 | 0,018±0,003 |
| **CONTROL 3** | 13,46±3,58 | 0,049±0,021 |
